# Unbiasing Retrosynthesis Language Models with Disconnection Prompts

**DOI:** 10.1021/acscentsci.3c00372

**Published:** 2023-07-05

**Authors:** Amol Thakkar, Alain C. Vaucher, Andrea Byekwaso, Philippe Schwaller, Alessandra Toniato, Teodoro Laino

**Affiliations:** † IBM Research Europe, Saümerstrasse 4, 8803 Rüschlikon, Switzerland; ‡ National Center for Competence in Research-Catalysis (NCCR-Catalysis), 8093 Zürich, Switzerland

## Abstract

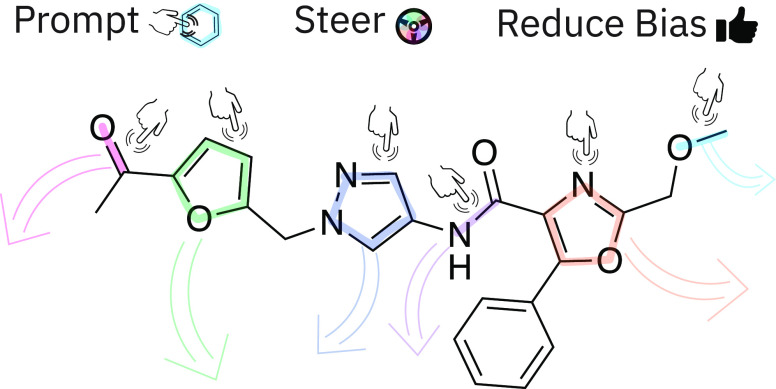

Data-driven approaches to retrosynthesis are limited in user interaction, diversity of their predictions, and recommendation of unintuitive disconnection strategies. Herein, we extend the notions of prompt-based inference in natural language processing to the task of chemical language modeling. We show that by using a prompt describing the disconnection site in a molecule we can steer the model to propose a broader set of precursors, thereby overcoming training data biases in retrosynthetic recommendations and achieving a 39% performance improvement over the baseline. For the first time, the use of a disconnection prompt empowers chemists by giving them greater control over the disconnection predictions, which results in more diverse and creative recommendations. In addition, in place of a human-in-the-loop strategy, we propose a two-stage schema consisting of automatic identification of disconnection sites, followed by prediction of reactant sets, thereby achieving a considerable improvement in class diversity compared with the baseline. The approach is effective in mitigating prediction biases derived from training data. This provides a wider variety of usable building blocks and improves the end user’s digital experience. We demonstrate its application to different chemistry domains, from traditional to enzymatic reactions, in which substrate specificity is critical.

## Abstract 2

Prompt-driven chemical language modeling empowers chemists to make more diverse and creative recommendations for retrosynthesis, reducing bias, and improving the end user digital experience.

## Introduction

**Figure 1:**
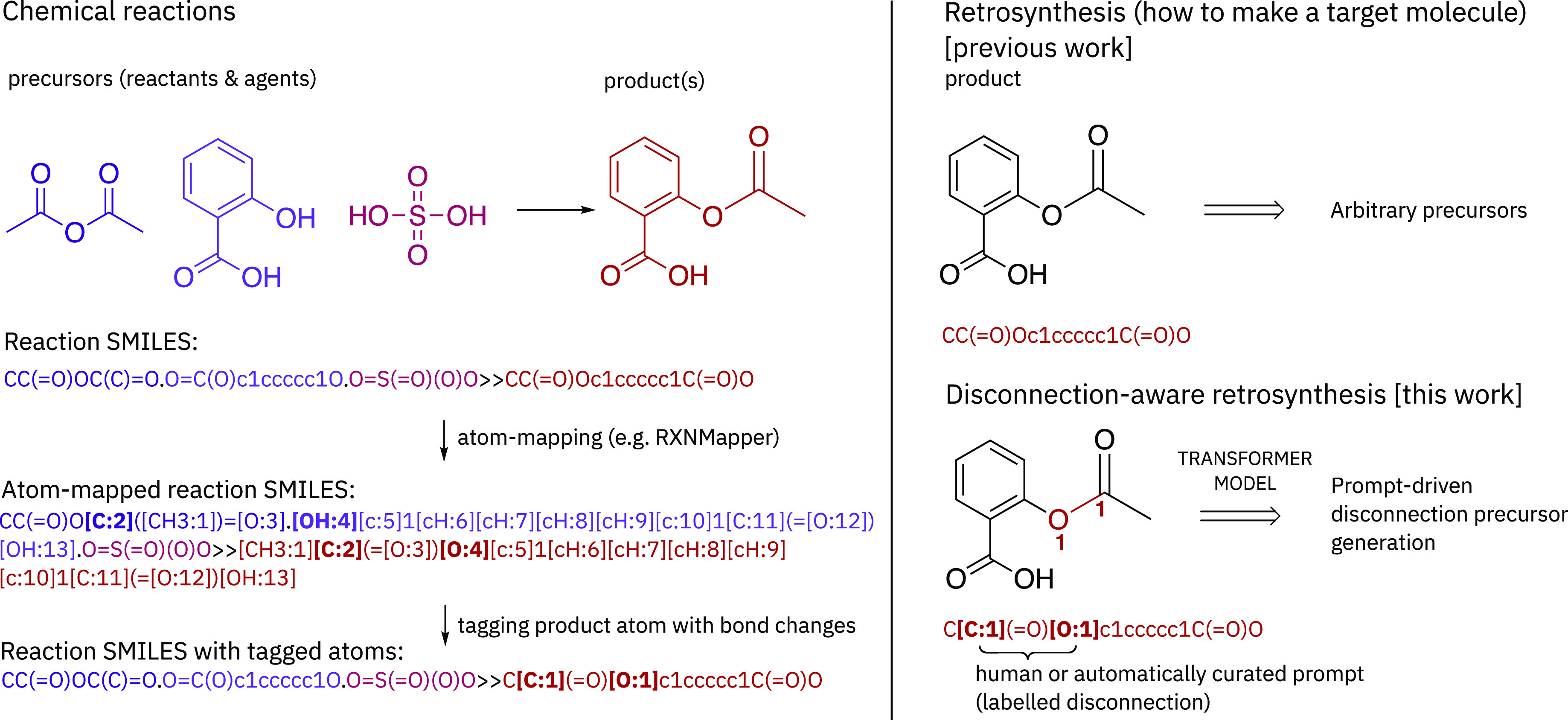
Left: Overview of the processing pipeline of the chemical reactions is represented as SMILES. The steps taken to tag the product atoms using atom-mapping notation are outlined. Right: A single-step retrosynthesis (upper) is compared with prompt-driven disconnection-aware retrosynthesis (lower).

Retrosynthesis is the task of determining the optimal sequence of steps required to synthesize a given molecule of interest starting from readily available building blocks. It was Corey et al. in the 1960s^1^ who pioneered the digitization of the process, followed by a range of approaches from heuristics on the basis of expert systems^2−4^ to data-driven deep learning.^5−10^ When performed by domain experts, single-step retrosynthetic analysis, i.e., the breakdown of a target product into its constituent set of precursors, can be seen as a two-step process. First, the expert identifies a suitable site of disconnection by considering the competitiveness of forming that specific chemical bond (Figure 1) across all others present. Thereafter, the attention focuses on choosing an optimal transformation on the basis of chemo-, regio-, and stereoselective considerations while optimizing yields, sustainability, and costs. Although the choice of a disconnection site should be based solely on the downstream synthetic route, it is frequently heavily influenced by the practitioner’s chemistry knowledge. The same is true for data-driven methods that incorporate the inherent chemical reactivity bias of training data sets.

Among the different computer-assisted planning schemes for chemical synthesis,^11^ deep-learning-based approaches using natural language processing (NLP) have become popular^8,9,12,13^ thanks to their high prediction accuracy,^14^ ease of adoption,^15^ seamless extension to novel reaction classes,^16^ and application to a wide range of digital chemistry tasks.^17−19^ Overall, language models offer the great advantage of learning the rules governing chemical transformations directly from raw data^20^ instead of requiring the explicit encoding of humanly crafted logic. Commonly relying on the use of the Transformer architecture^21^ and the simplified molecular-input line-entry system (SMILES) notation,^22,23^ NLP models treat the prediction of chemical species as a translation task. Given a target molecule, language models suggest the best set of precursors (i.e., reactants, and possibly other reagents) as the translation’s outcome(s), with the possibility to generate multiple such sets. Nevertheless, similar to the human bias of favoring more familiar reaction classes, data-driven models exhibit a bias inherited from reaction data sets used for training. This leads to a poor diversity of predictions, with the proposed retrosynthetic disconnections often belonging to the most abundant reaction classes in training data sets, such as protection/deprotection or oxidation/reduction for those derived from patents.^24,25^ The inherent bias in these recommendations conceals the broader options encompassed by multiple disconnection sites, thereby restricting the variety of precursors, which in turn reduces the effectiveness of any computer-aided synthesis plan. In their current format, data-driven approaches to retrosynthesis afford little control to users in steering the prediction of the translation’s outcome(s). Here, we explore the use of prompt-based learning to mitigate model biases inherited from training data sets. Prompt-based learning is an emerging paradigm in computer science that opens up the possibility of enhancing interactions with AI-based systems to guide inference along directions determined by the input prompt. GPT-3 and DALL-E, which are capable of generating human-like text and realistic images given a set of human- or machine-generated inputs,^26,27^ are a few examples of the success of this technology.

Compared with previous uses of language models for the prediction of chemical reactions, we introduce, for the first time, the concept of steering with human or machine inputs of the chemical predictions in retrosynthesis inference tasks (Figure 1). We introduce prompts that specify the disconnection site to integrate deep learning algorithms with domain knowledge and experience. The disconnection prompts can be human- or machine-labeled, and they are used to steer the translation of a product into a set of precursors, thus leading to an improvement in predicted reaction class diversity exceeding 100%. We validate this novel scheme both with traditional chemical transformations and enzymatic reactions for biocatalysis. The results demonstrate a 39% performance improvement over baseline models and confirm the possibility to use human-in-the-loop approaches across chemical synthesis and biocatalysis for an improved retrosynthesis experience. The use of prompt-based learning is demonstrated to be an easy to adopt and very effective approach for mitigating biases inherited from training data sets. Ultimately, the use of prompts has the potential to open up new training data acquisition campaigns that will facilitate performance improvements, diversity, and interactivity of future retrosynthesis models.

## Results and Discussion

**Figure 2:**
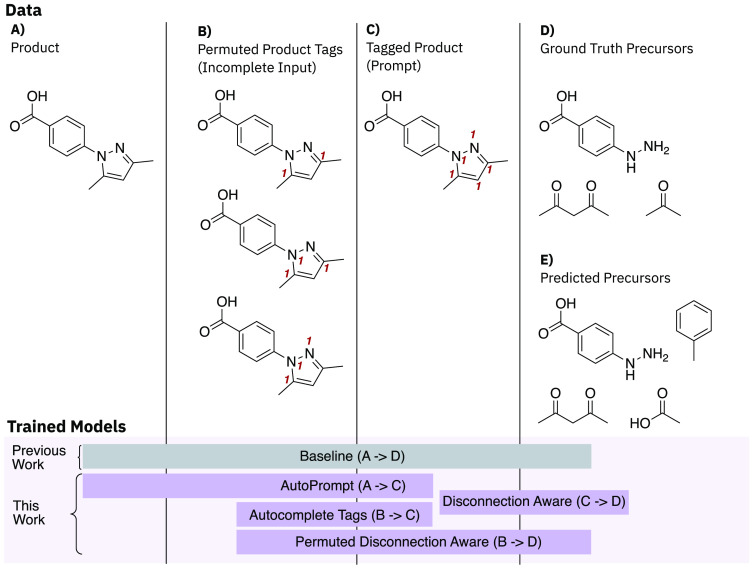
Overview of the experiments conducted and models trained illustrated with an example of a heterocycle formation.

Chemical language modeling predominantly makes use of sequence-to-sequence Transformer architectures. When applied to retrosynthesis, a SMILES string representing the molecule of interest is used as input, and the model generates the set of precursors (reactants and reagents) as the translation’s outcome(s). Henceforth, we consider the “Molecular Transformer” (MT) developed by Schwaller et al.^9,14^ as a reference point for development of our prompt-driven *disconnection aware* model (Figure 2) and refer to it as the *baseline* model. The *baseline* model is not directly used for prompt-based learning nor is it able to accept guiding prompts in its input. To overcome this problem, we developed the *disconnection aware* model that can direct the outcome of a translation using an additional input prompt, as opposed to the *baseline* model, which generates predictions toward certain disconnections solely on the basis of the underlying probability distribution of chemical transformations in training data sets. To compare the *disconnection aware* model with that of the *baseline*, we utilized the disconnection information to select precursors generated by the *baseline* model that matched the chosen disconnection site. The use of guiding prompts resulted in a 39% increase in accuracy (i.e., the *baseline* model could not produce suitable precursors matching the disconnection site in 39% of cases) and was further verified by examining the TopN accuracy (Supporting Information), for which we did not see an improvement across an increasing number of predictions (*N*). As the TopN accuracy does not increase for the *baseline* model, this implies that suitable sets of precursors could not be predicted for the desired disconnection site. In addition, we observed a 100% (2-fold) increase in reaction class diversity of the *disconnection aware* model as opposed to the *baseline*.

### Disconnection Aware Retrosynthesis

The original MT does not natively support user-defined prompts or the tokens used for their representation. It follows that, across all data sets examined, the *baseline* model was not predictive when used in combination with inputs containing tagged disconnection sites (prompts). Therefore, we trained the *disconnection aware* model using product SMILES containing labeled prompts in the form of atom tags, as shown in Figure 2C, as input and using the ground truth precursors as labels (Figure 2D).

**Figure 3:**
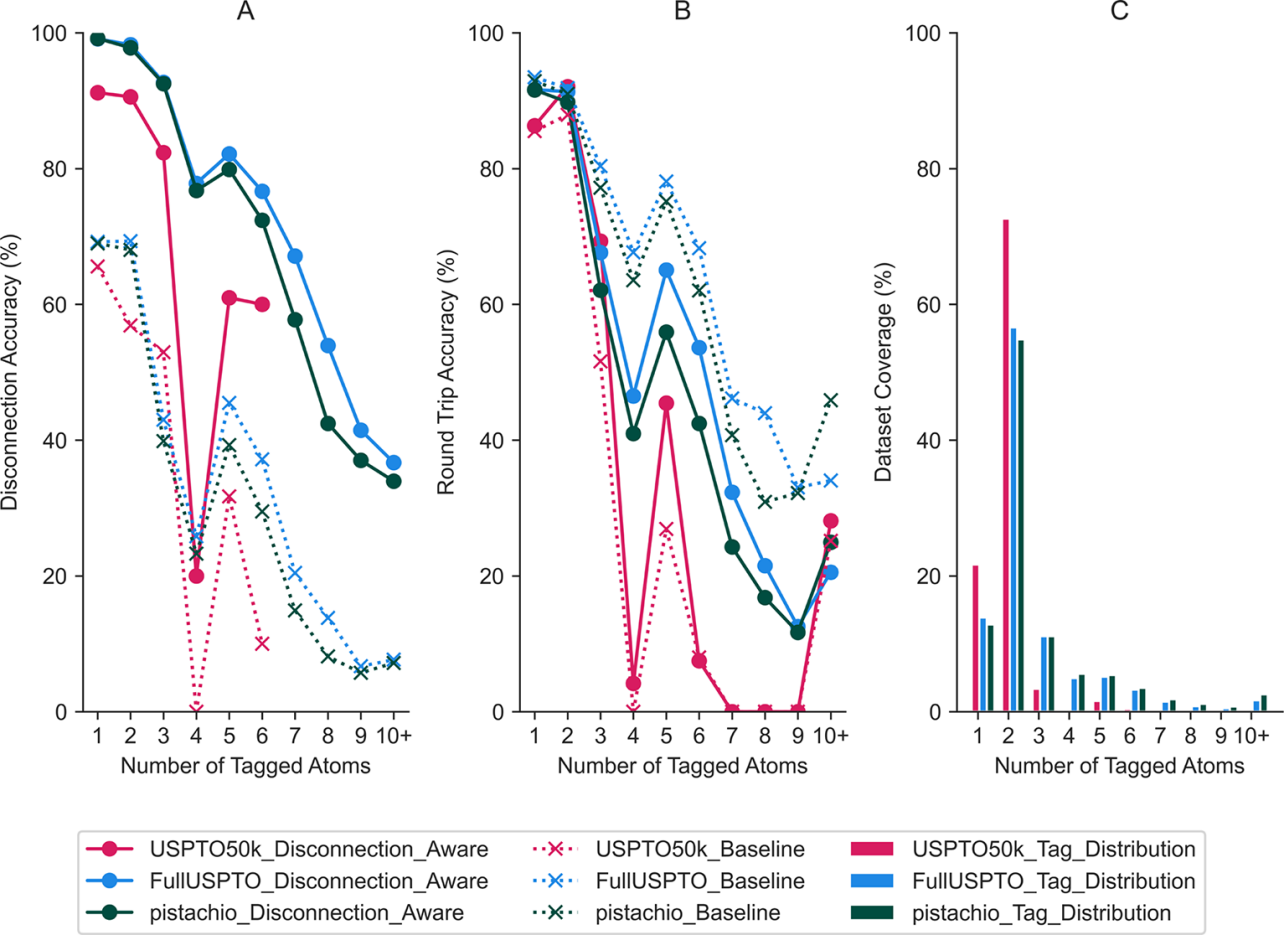
(A) Comparing the *disconnection aware* model against the *baseline* across the number of atoms that constitute the disconnection site. The disconnection accuracy reflects the ability of the model to predict the correct bond changes, for which we observe that the *disconnection aware* model outperforms the *baseline*. (B) The round trip accuracy refers to the ability to regenerate the required product from the precursors predicted by the *baseline* and *disconnection aware* models, respectively. The baseline model exhibits slightly higher round trip accuracy, although the precursors predicted are different from the ground truth, as evidenced by the lower disconnection accuracy. The number of tagged atoms has been recalibrated to the predicted bond changes. (C) Tag distribution across the Pistachio, USPTO, and USPTO50k data sets. The performance of the models is seen to correlate with the availability of training data for a given number of tags.

Of primary interest were two metrics: first, the round trip accuracy as developed by Schwaller et al.^9^ to determine whether the desired product could be regenerated from the predicted precursors, and second, the disconnection accuracy, to determine whether the predicted precursors corresponded to disconnection at the user-defined position (Figure 3). The disconnection accuracy was computed by reconstructing the reaction using the input product and predicted precursors from which the disconnection site could be determined. If the disconnection site matched that in the test set, then there would be a positive impact on the disconnection accuracy. In addition, the accuracy metric was reported across the number of tagged atoms rather than taking an overall TopN accuracy. This metric offers a more granular understanding of the types of disconnections present in the data set, how well they are reproduced, and whether there is a tendency for the model to be biased toward a given disconnection type—information that cannot be obtained from the TopN metric alone.

We determined that the *disconnection aware* model outperformed the *baseline* model by an average of 39% across all number of atom tags and data sets with respect to the disconnection accuracy (Figure 3A). This demonstrates that the *disconnection aware* model is better able to reconstruct reactions corresponding to a user-specified disconnection than the *baseline* model. In addition, we observed that the disconnection and round trip accuracies correlate with the availability of training data for a given number of atom tags (Figure 3A,B). Most notable is the performance drop when the number of tags equals four because of no examples being present in the USPTO50k data set we processed. However, despite there being no training examples containing four atom tags in the USPTO50k data set, the model was still able to recover 20% of the reactions, thereby demonstrating the model can extrapolate to unseen reactions. Similarly, low performance was observed when the number of tags was greater than five because of a decrease in the availability of training data.

**Figure 4:**
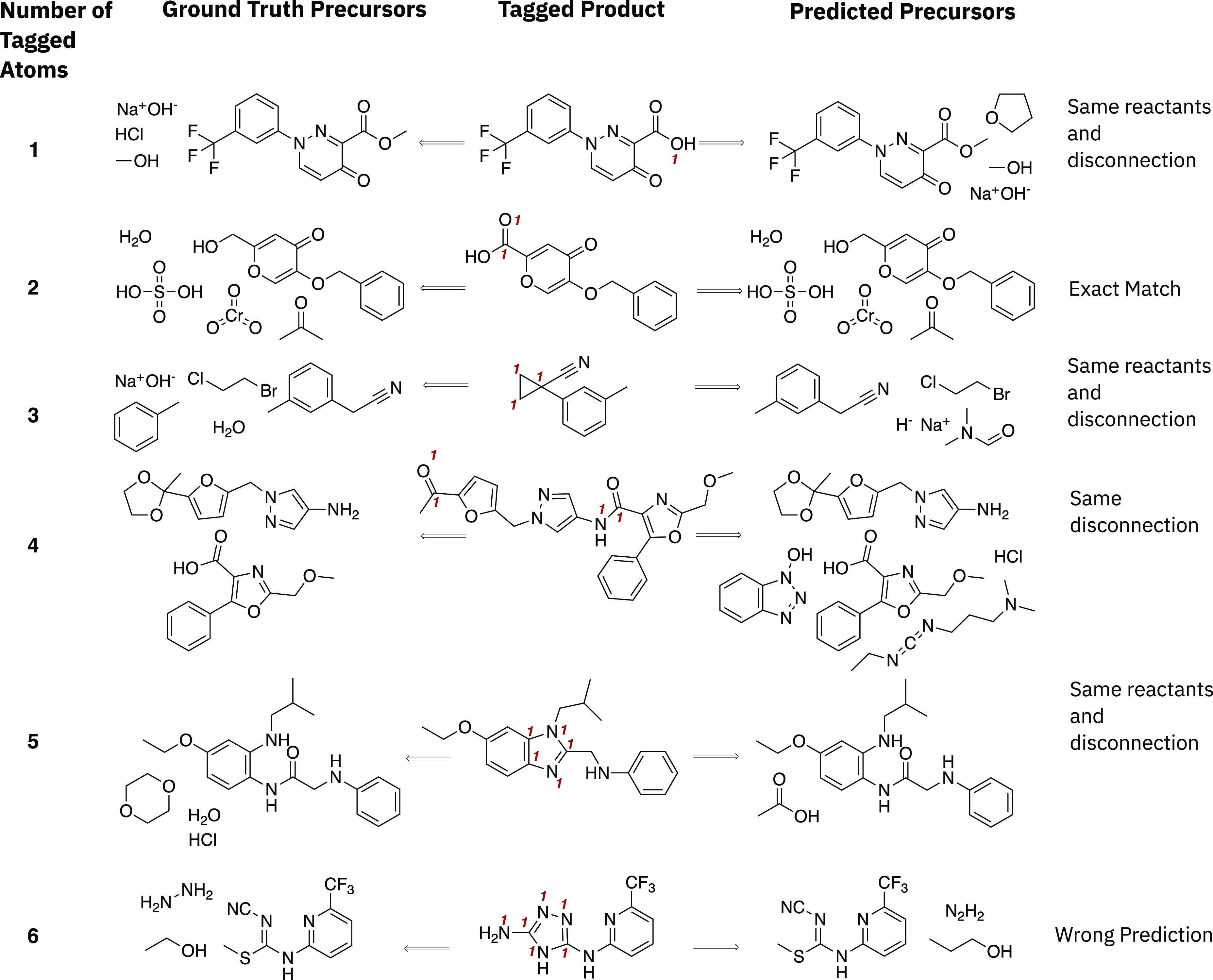
Examples comparing the predicted precursors from the *disconnection aware* model to the ground truth, as extracted from the test set. Different sizes of disconnection site are shown as labeled by atom tags. The *disconnection aware* model is able to target specific sites of disconnection and produce appropriate precursors in line with the ground truth. Although the major components are predicted according to the ground truth in most cases, some variability exists in the reagents that are predicted for the transformation.

Furthermore, we observed a bias in the patent data toward disconnection sites involving two atoms (i.e., one bond change). These findings are in line with that observed from surveys conducted by Böstrom et al. and others studying the frequency of the types of reactions that are reported in both public and proprietary data sets.^28,29^ Despite the bias toward reactions involving one bond change, the *disconnection aware* model can produce predictions across all number of atom tags, including those for heterocycle formations, as shown in Figure 4. Unlike previous approaches developed for predicting ring disconnections, which afford the user no control over which ring system to disconnect,^30^ the *disconnection aware* model allows chemists to target specific ring systems via the tagging mechanism.

#### Assessing the Impact of Reagents

While the correct disconnection and reactants as the ground truth are often predicted, there remains a set of variable species, so-called reagents, that may not match the ground truth (Figure 4). This can be explained by considering the role of the different chemical species in sequence-based model training. Usually, the major components (i.e., those that contribute atoms to the product) can be atom-mapped.^20,31^ Thus, these must be present for the reaction to occur. The unmapped components correspond to solvents, bases, additives, catalysts, etc., which can be variable. For the disconnection aware retrosynthesis task, we performed two different analyses: one including all species and the other excluding unmapped species. The results when removing the unmapped species showed an increase in the percentage of predictions matching the ground truth from 14% to 41% for the USPTO data set. Comparable performance increases were seen across the other data sets examined. Although the percentage of predictions matching the ground truth increased significantly, both round trip and disconnection accuracy remained comparable with the baseline values regardless of the removal of the unmapped species. Thus, we can conclude that the differences in predictive performance do not arise from variations in the prediction of species that contribute to the product but are a result of the unmapped species that are variable. We also observed that this has negligible influence on the ability to predict the correct product using the forward model, most likely because of the sparse sampling of the chemical space in data sets used for training.

### Autocomplete Tags—Accounting for Incomplete Prompts

**Figure 5:**
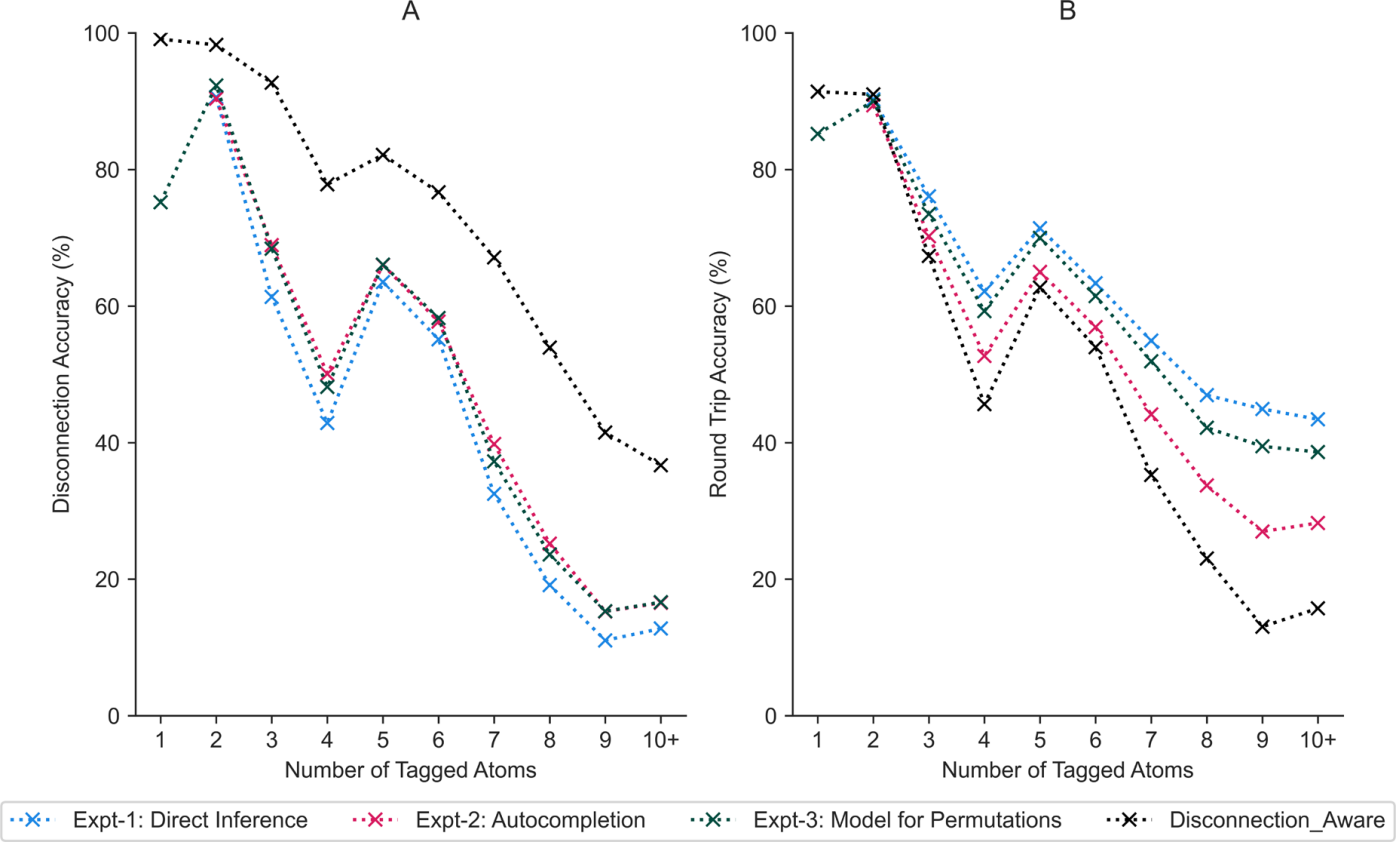
(A) The *disconnection aware* model with complete tags remains the most well-performing for disconnection accuracy, while the approaches used for dealing with incomplete user input exhibit comparable performance. (B) The round trip accuracy across the number of tagged atoms is comparable up to a number of tags equaling six; thereafter, a deviation occurs, whereby the *disconnection aware* model decreases in performance, and Expt-1, inference directly from incomplete user input, maintains almost 50% round trip accuracy.

Given that the transformer-based models trained in this study are created to be interactive, the model should be robust to user input. This means that a user may enter a set of incomplete tags. To simulate this event, we permuted the tags up to a preset limit of four tags, as shown in Figure 2. Three experiments were conducted to determine which approach to take when dealing with incomplete user input. First (Expt-1), we inferred directly from incomplete input using the *disconnection aware* model. Second (Expt-2), we autocompleted the disconnection site using the model termed *autocomplete tags* followed by inference with the *disconnection aware* model. Third (Expt-3), we inferred using a variation of the disconnection aware model trained using permuted tags as input and the ground truth precursors as training labels, aptly named the *permuted disconnection aware* model (outlined in Figure 2). The results for these approaches are shown in Figure 5 and are compared with the *disconnection aware* model with complete tags for the USPTO data set. Comparable results were obtained for the Pistachio and USPTO50k data sets (as shown in the Supporting Information).

All three strategies outlined for handling incomplete atom tags showed comparable performance for disconnection and round trip accuracy when the number of tagged atoms is below six. Beyond six tagged atoms, a deviation occurs in the round trip accuracy, which favors the direct inference from incomplete input using the *disconnection aware* model (Expt-1). Furthermore, all three strategies exhibit a disconnection accuracy lower than that of the model given complete tags. Therefore, the *disconnection aware* model shows a certain level of robustness to incomplete disconnection sites, albeit with a slight decrease in disconnection accuracy, and a comparable round trip metric.

### Prompt-Driven Steering of Retrosynthesis Prediction

**Figure 6:**
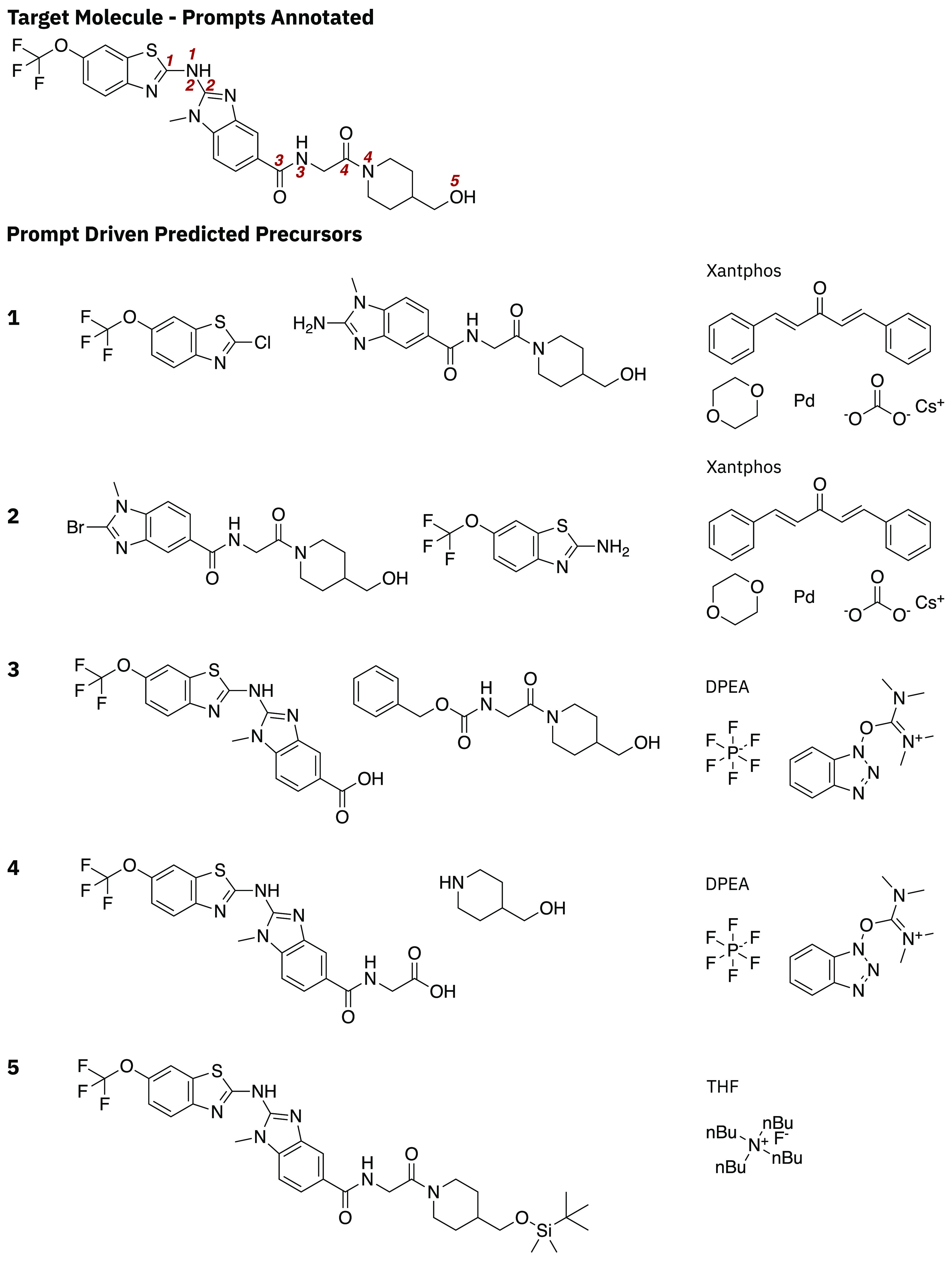
Human-curated prompts for steering the retrosynthetic prediction. Prompts are labeled on the target molecule, and the predictions are listed with the corresponding label (left column).

Having demonstrated that the *disconnection aware* model outperforms the *baseline* Molecular Transformer and can reproduce the required set of precursors when prompted with a label tagging the disconnection site, we studied whether valid sets of precursors would be generated for arbitrary disconnections. In doing so, we investigated whether the *disconnection aware* model could be steered to produce alternative outputs for the same molecule, an example of which is shown in Figure 6. The results show that the *disconnection aware* model is capable of distinguishing between similar sites of disconnection and producing valid sets of precursors, as shown for sites labeled 1 and 2 in Figure 6. The ability to arbitrarily specify sites of disconnection and prompt the model toward alternative outputs opens up new avenues for chemical language modeling, which serve to facilitate user interaction with the model. For example, current models are criticized for their lack of diversity in the choice of disconnection site, which results in predictions with identical building blocks and a wide variety of reagents. “Human-in-the-loop” prompting of the *disconnection aware* model is an effective strategy to mitigate this behavior by enabling the steering of the model toward alternative reaction classes, therefore improving prediction diversity, as shown in Figure 6. Furthermore, as a consequence of uncovering the ability to steer model predictions, we propose that prompting the *disconnection aware* model can be used to investigate chemical language modeling more generally.

The prompt-driven approach offers significant advantages over methods that require the encoding of chemical reaction rules. To illustrate this, consider Figure 6. For each of the five disconnections that can be specified by a user prompt, a rule-based approach would require specification of a reaction rule. Furthermore, the number of reaction rules that must be specified scales with the number of different reaction types and their specific atomic environments. For example, Figure 6.1 could be carried out with a Br in place of the Cl; therefore, at least two rules must be specified for the transformation. Whereas in the prompt-driven approach, the model is able to learn the patterns directly from the available reaction data without prior knowledge of the reaction rules. A further problem associated with reaction rules is that they are applied to all substructures within the query molecule matching the reaction rule, thus leading to a diverse range of unwanted outcomes. In the prompt-driven approach, the user is able to specify the disconnection site from which predictions should be made, and the model is able to selectively generate precursors starting from the user-defined location.

### Improved Class Diversity for Automatic Retrosynthesis

**Figure 7:**
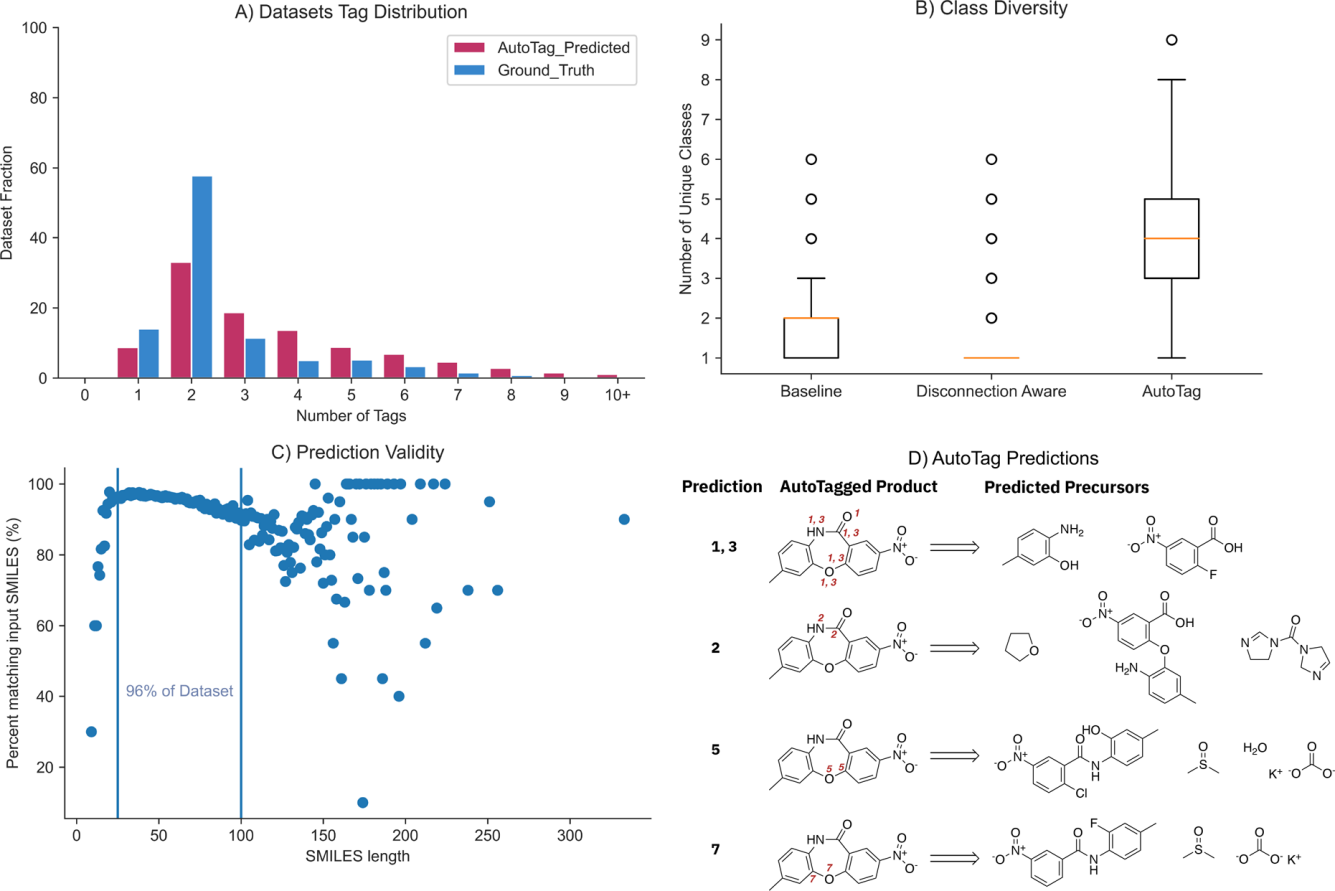
(A) Distribution of predicted atom tags as a function of the fraction of the data set compared with the ground truth. Disconnections sites consisting of three or more tagged atoms are oversampled by the *AutoTag* model, although the general distribution follows the same trend as the ground truth. (B) Class diversity corresponds to the number of unique reaction classes predicted per sample across the top 10 predictions. At least a 2-fold improvement in class diversity is observed when using the *AutoTag* workflow for automatic retrosynthesis. The *disconnection aware* model predicts only one reaction class by specific disconnection site labeling. (C) The percentage of predictions across the top 10 matching the input as a function of SMILES length. In 96% of cases, the errors fall within an acceptable range. (D) Predictions obtained using the *AutoTag* workflow for a given sample taken from the test set. The prediction rank is used for labeling the disconnection site for the figure.

While the focus thus far has been on “human-in-the-loop” retrosynthesis, the *disconnection aware* model can be adapted to cases where human interaction is not possible or not desired, such as automatic or multistep retrosynthesis prediction. We show that using a model trained to automatically label disconnection sites, which we call *AutoTag*, followed by inference with the *disconnection aware* model improves predicted reaction class diversity by at least a factor of 2 (100% increase) for the USPTO data set in comparison with the *baseline model* (Figure 7B).

The number of tags predicted by the *AutoTag* model follows approximately the same distribution as the ground truth data and inherits the same type of disconnection bias. Notably, the model tends to predict disconnection sites that are larger than those represented in the ground truth data set, as shown in Figure 7A. Although performance decreases with the size of the disconnection site, as represented by the number of tagged atoms (Figure 3), we found that SMILES length, thus the size of the molecule, can be a detrimental factor (Figure 7C). As the number of predictions for a given SMILES increases (TopN), the performance of the *AutoTag* model deteriorates, which concerns the ability to reproduce the original input. However, we found that for 96% of the reactions in the data sets examined (SMILES length between 25 and 100 characters) the error was within an acceptable range for the top 10 predictions, as shown in Figure 7. Figure 7D shows selected examples of the *AutoTag*-predicted disconnection sites and precursors for a given molecule (more details for all predictions are available in the Supporting Information).

### Extension to Enzyme Catalysis

**Figure 8:**
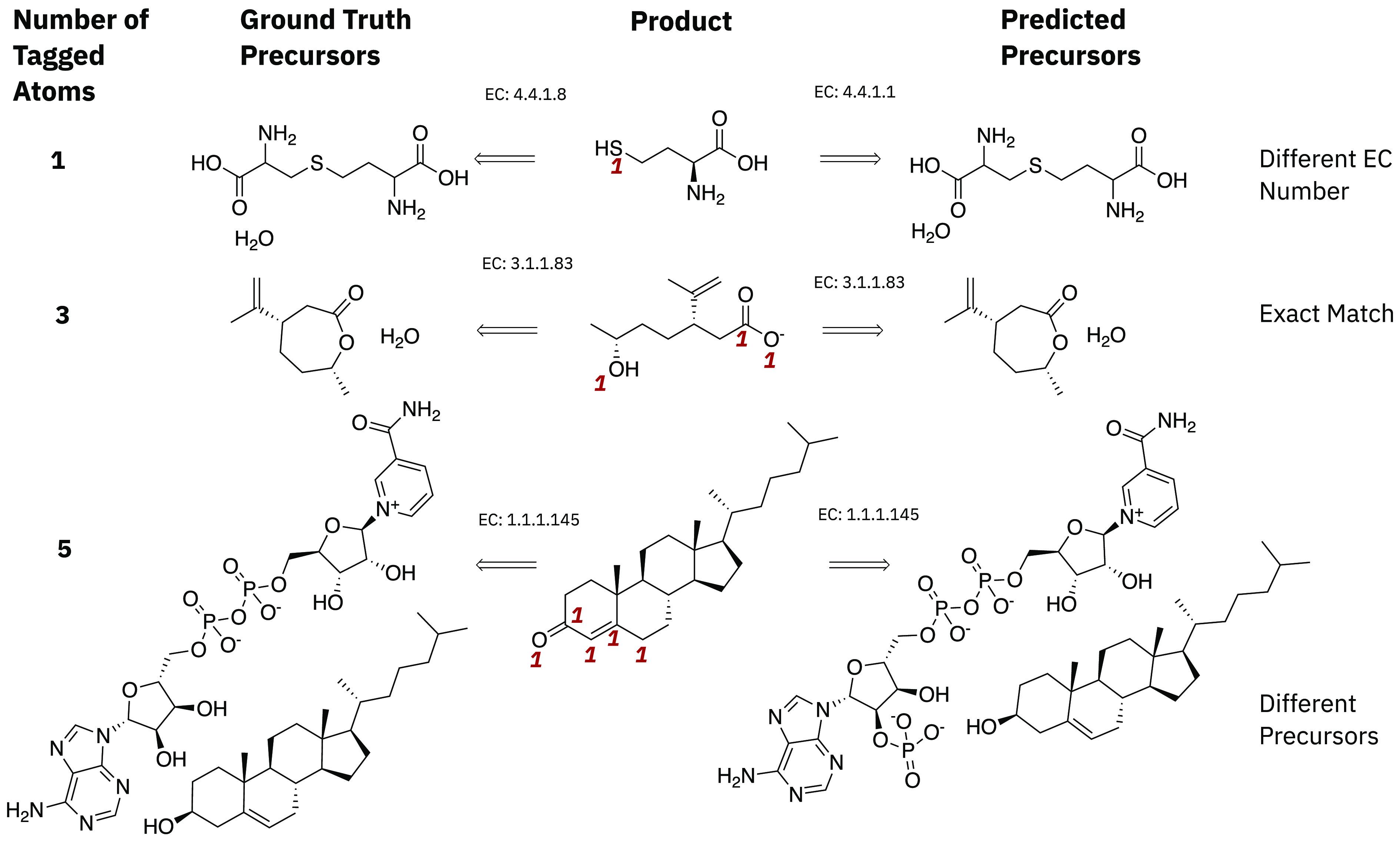
Examples from the enyzme test set with different numbers of tagged atoms.

Probst et al.^32^ and Kreutter et al.^33^ have previously demonstrated that sequence-to-sequence Transformer models can be applied to enzymatic reactions, thus broadening the scope of language models in chemistry to biocatalysis. Here, we extended the disconnection-aware retrosynthesis approach by applying our atom-tagging procedure to enzymatic reactions using the ECReact data set^32^ and, subsequently, to training models following the approach described by Probst et al.^32^ A key distinction in the treatment of the enzymatic reaction SMILES was the inclusion of the enzyme *EC* number. To tag the disconnection site, the enzyme *EC* number was omitted to facilitate atom-mapping with RXNMapper.^20^ However, it was reintroduced into the SMILES reaction for training and inference tasks. The trained model achieved an average disconnection accuracy of 79%, thereby exceeding the performance on the Pistachio, USPTO, and USPTO50k data sets, and an average round trip accuracy of 52% across all number of tagged atoms. Figure 8 shows a few examples of some of the predictions from the *disconnection aware* model trained on enzymatic data.

## Conclusion

This study introduces for the first time the use of a prompt-driven language model for mitigating prediction biases inherited from trained data. We demonstrate that a prompt-driven *disconnection aware* model is very effective in steering the outcome of a sequence-to-sequence language model to achieve up to 73% prediction accuracy, which is a 39% performance improvement over the baseline model when trained on the same data set. Similar improvements extend to enzymatic data, for which we achieve an overall 79% prediction accuracy.

We also extend the *disconnection aware* model to be compatible with automatic or multistep predictions by utilizing a model trained to automatically label the disconnection site, which we term *AutoTag*. The use of the *disconnection aware* model in combination with the *AutoTag* model demonstrates an improved performance on reaction class diversity by a 2-fold factor (100% increase), thereby providing a very effective solution to overcome criticisms toward the reduced diversity performance of traditional Molecular Transformer architectures.

This work marks a shift from previous ideas exploring the use of sequence-to-sequence Transformer architectures for chemical language modeling. Similar to the approach followed by human experts when performing a retrosynthetic analysis, the use of a prompt-based language guides the inference of reaction prediction models toward those reactions that involve the creation of a selected bond or set of bonds. The use of prompt-based language models opens up the possibility of using human-in-the-loop approaches across chemical synthesis and biocatalysis for an improved retrosynthesis experience. Finally, the use of the human-designed prompts opens the door to systematic improvements in retrosynthetic planning tools thanks to the effective decision-making combination of expert knowledge and deep learning.

## Methodology

### Data and Preprocessing

The United States Patent Office extracts comprised of the Pistachio data set (2022Q1) from NextMove software,^24^ the publicly available subset USPTO extracted by Lowe,^25,34^ and the USPTO50k subset from Schneider et al. were used in this study.^35^ An extension of the method to ECReact containing enzyme data as curated by Probst et al. was used for the enzymatic model.^32^ Each data set consisted of reactions in the SMILES notation, atom-mapped using RXNMapper, as shown in Figure 1.^20^ Atom-mapping was required in order to determine which atoms and bonds had changed in the reaction for subsequent labeling of the disconnection site. The data sets were preprocessed using an internal preprocessing pipeline to filter for reactions with one product, between 2 and 10 reactants, and token constraints as specified in the Supporting Information. Reactions with no tagged atoms were removed because they imply no atom bond changes were detected; therefore, no reaction occurred. Reactions with >10 tagged atoms were also removed. The data sets were split into training, validation, and test sets in a 90:5:5 ratio, respectively, and tokenized using a regex pattern, as described by Schwaller et al.^14^

### Prompt Generation—Extracting Atom Tags

Prompts can be either human-curated or automatically extracted if relevant training data is present. In this study, we automatically extracted a prompt from reactions’ SMILES by identification of the changed atoms and bonds in the product. The automatically extracted prompts were solely used for model training in place of human-curated prompts. The prompt corresponds to the site of disconnection and is labeled in the product’s SMILES by identification of the atoms for which the bond order differed between reactants and products for a given atom-mapped reaction’s SMILES. Atom-mapping was removed from the precursors and products after the disconnection site was identified, and atom tags were introduced to the product using the SMARTS notation [*:1],^36^ where ‘*’ resembles any atom to signify atoms to be changed, as shown in Figures 1 and 2. The pseudocode is outlined in the Supporting Information. The prompt-labeled data containing atom tags were used to train the *disconnection aware* model, as shown in Figure 2C, using the ground truth precursors as labels (Figure 2D). We additionally tested the *baseline* model to determine whether prompts were tolerated.

### Tag Permutation

Given that the *disconnection aware* model is designed to introduce a human-in-the-loop component for retrosynthesis, we took into consideration the possibility that there may be incompletely tagged disconnection sites. Therefore, tag permutations were generated for all products to emulate incomplete user input. Products with the number of tags equal to one were omitted, given that no permutations are possible. All remaining products were permuted up to a preset limit of four tagged atoms upon sampling to avoid a too large number of permutations. For example, a five-membered ring system would have up to four of the atom tags permuted, an example of which is shown in Figure 2. The permuted data, as shown in Figure 2B, was used as input to train a model using the ground truth precursors as labels (Figure 2D), the so-called *permuted disconnection aware* model. We additionally used the permuted data to test whether the *baseline* and *disconnection aware* models could tolerate incomplete input.

### Tag Completion

An alternative approach to deal with incompletely tagged disconnection sites is to introduce an autocompletion model as a step between user input and prediction of a set of precursors, henceforth referred to as the *autocomplete tag* model. The model was trained as a supervised learning task, where the input was our generated set of permuted atom tags that emulate incomplete user input. The corresponding labels consisted of the completed atom tags as extracted automatically from the reaction SMILES.

### AutoPrompt—Automatic Labeling of Disconnection Sites

Although the *disconnection aware* model was originally intended to be used in a human-in-the-loop manner, we observed notable improvements to predicted reaction class diversity arising from the fact that multiple disconnection sites can be labeled, thus generating alternative precursor sets. This led us to develop a mechanism by which the *disconnection aware* model can be used for automated retrosynthesis. We introduced the *AutoTag* model trained on the products’ SMILES (Figure 2A) as input and the atom-tagged SMILES (Figure 2B) as labels. To mitigate potential data leakage when combining the *AutoPrompt* and *disconnection aware* models, we used the same training data set for the two models. The differences in training arise from the different input and labels used.

### Training

All models used supervised learning and a seq-seq Transformer architecture, as implemented in the OpenNMT-py library version 1.0.0.^37,38^ The Transformer models for the enzyme data set were trained using the adaptation and commands outlined by Probst et al.^32^

### Metrics and Analysis

Evaluation was performed on the hold-out test set using the round-trip metric, as established by Schwaller et al.,^14^ in addition to metrics defined in this manuscript to evaluate the disconnection accuracy. All evaluations were conducted after standardization of the reaction components. The disconnection accuracy was determined by running a retrosynthetic translation to obtain the set of predicted precursors. The top 1 predicted precursor was then fed into a forward reaction prediction model trained on the Pistachio data set,^24^ in this case, the default model used by the Molecular Transformer, and returned a predicted product.^9^ The predicted product was evaluated to determine whether it matched the ground truth, which constitutes round trip accuracy. By extension, the disconnection accuracy was computed by reconstituting the reaction using the top 1 predicted precursors and corresponding predicted product from the forward model, remapping with RXNMapper,^20^ and recomputing the tagged atoms, thus the disconnection site. Evaluation was then performed to determine whether the disconnection site obtained from the prediction matched the ground truth. For a complete set of models trained and evaluations, refer to the Supporting Information.

Evaluations were conducted to examine the TopN accuracy across a variety of metrics in addition to examining the performance across the number of tagged atoms.

## Data Availability

Code to identify the disconnection sites and train the models can be found on our GitHub repository: https://github.com/rxn4chemistry/disconnection_aware_retrosynthesis. The *disconnection aware* model can be used for multistep retrosynthesis on: https://rxn.res.ibm.com/. The US patent extract text mined by Lowe is publicly available and can be processed using code on our GitHub repository.^25,34^ We additionally provide a preprocessed and labeled set of data obtained from the US patent extracts text mined by Lowe: https://zenodo.org/record/7101695#.Yzr96SFBx4A.

## Supporting Information Available

Additional figures and results, including discussion, data and preprocessing, constraints on reactions, prompt generation for the extraction of atom tags, tag completion, OpenNMT model training with metrics and experiments, and improved class diversity (PDF)
