# Supplementary material for: Unbiasing Retrosynthesis Language Models with Disconnection Prompts
Source: ACS Cent Sci. 2023 Jul 5;9(7):1488–98. doi: 10.1021/acscentsci.3c00372 (PMC10390024; doi:10.1021/acscentsci.3c00372)
Supplement: Supplementary file 1 — oc3c00372_si_001.pdf [file oc3c00372_si_001.pdf]

Supporting Information: Unbiasing Retrosynthesis Language Models with Disconnection Prompts

Amol Thakkar[1,2,*], Alain C. Vaucher[1,2], Andrea Byekwaso[1], Philippe Schwaller[1,2], Alessandra Toniato[1,2], and Teodoro Laino[1,2]

[1] *IBM Research Europe, Saümerstrasse 4, 8803 Rüschlikon, Switzerland*
[2] *National Center for Competence in Research-Catalysis (NCCR-Catalysis), Switzerland*
[*] *tha@zurich.ibm.com*

A. Results and Discussion

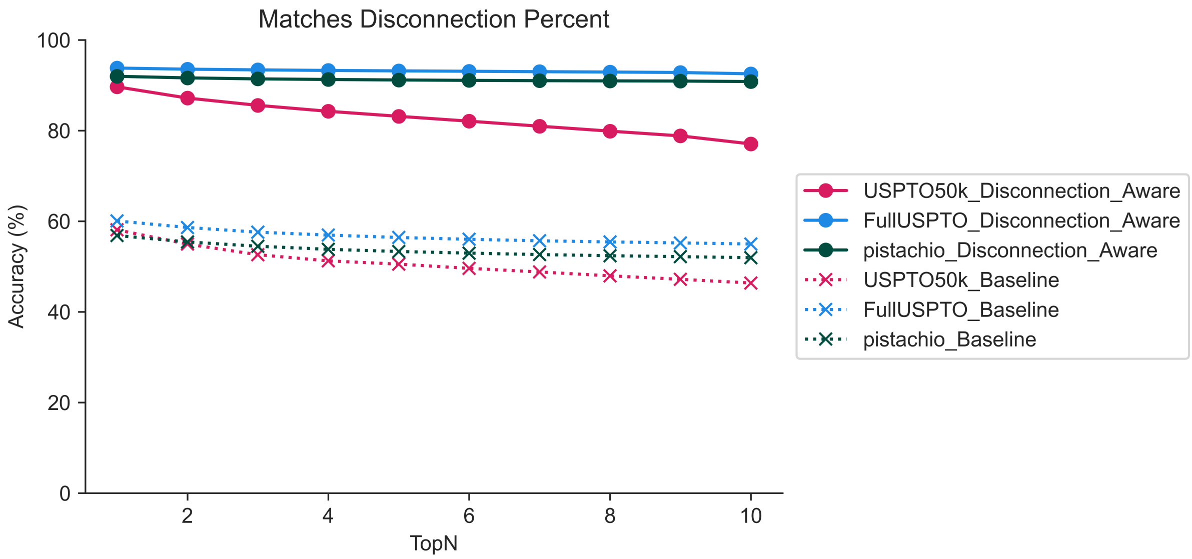

Figure S1: TopN accuracy for the ability to reproduce the disconnection site across the *disconnection aware* and *baseline* models, where N is the number of predictions. The disconnection site was determined by reconstituting the reaction with the predicted precursors and query product. If the predicted precursors could be obtained from the pre-labelled disconnection site, then the prediction would increase the topN accuracy metric. However, we observe that topN accuracy decreases for the *baseline* models. Thus, this shows that the predicted precursors correspond to a different disconnection site than that pre-labelled in the test set, and no suitable precursors are generated for the desired disconnection. In comparison, the *disconnection aware* models are consistently able to predict suitable sets of precursors with the exception of the 'USPTO50k' dataset, for which there is a decline in performance.

B. Data and Preprocessing

Table S1: Training, validation, and test set sizes for each dataset used in this study.

Dataset	Train	Valid	Test
ECReact (Enzyme)	42,042	2,277	2,334
USPTO50k	44,162	2,390	2,478
FullUSPTO	1,139,608	63,454	63,672
Pistachio	1,752,285	97,393	97,222

C. Constraints on Reactions

Table S2: Constraints placed on reaction SMILES during preprocessing. All 'reagents' were moved to the reactants before preprocessing, hence why min and max reagents equals zero.

Constraint	Value
minimum number of reactants	2
maximum number of reactants	10
maximum number of reactants tokens	300
minimum number of reagents	0
maximum number of reagents	0
maximum number of reagents tokens	0
minimum number of products	1
maximum number of products	1
maximum number of products tokens	200
maximum absolute formal charge	2

D. Prompt Generation - Extracting Atom-Tags

Algorithm 1 sketches the pseudo code used to prepare the training data for the model.

Algorithm 1 A function that converts the molecule objects for the precursors and product (typically in RDKit Mol format) to the format required for training the model. *all_atom_map_numbers* is a function returning the list of atom map indices present in the product object. *neighborhood* refers to the neighboring atoms and corresponding bond types.

Input:

precursors: Molecule object for the precursors, including atom mapping information

product: Molecule object for the product, including atom mapping information

Output:

precursors, product: a tuple containing the new molecule objects for precursors and product

```
transformed_atoms ← list()

for index ∈ all_atom_map_numbers(product) do
   precursors_atom ← precursors[index]
   product_atom ← product[index]
   if neighborhood(precursors_atom) ≠ neighborhood(product_atom) then
      transformed_atoms.append(product_atom)
   end if
end for

for atom ∈ precursors do
   atom.mapping = 0
end for

for atom ∈ product do
   if atom ∈ transformed_atoms then
      atom.mapping = 1
   else
      atom.mapping = 0
   end if
end for
```

E. Tag Completion

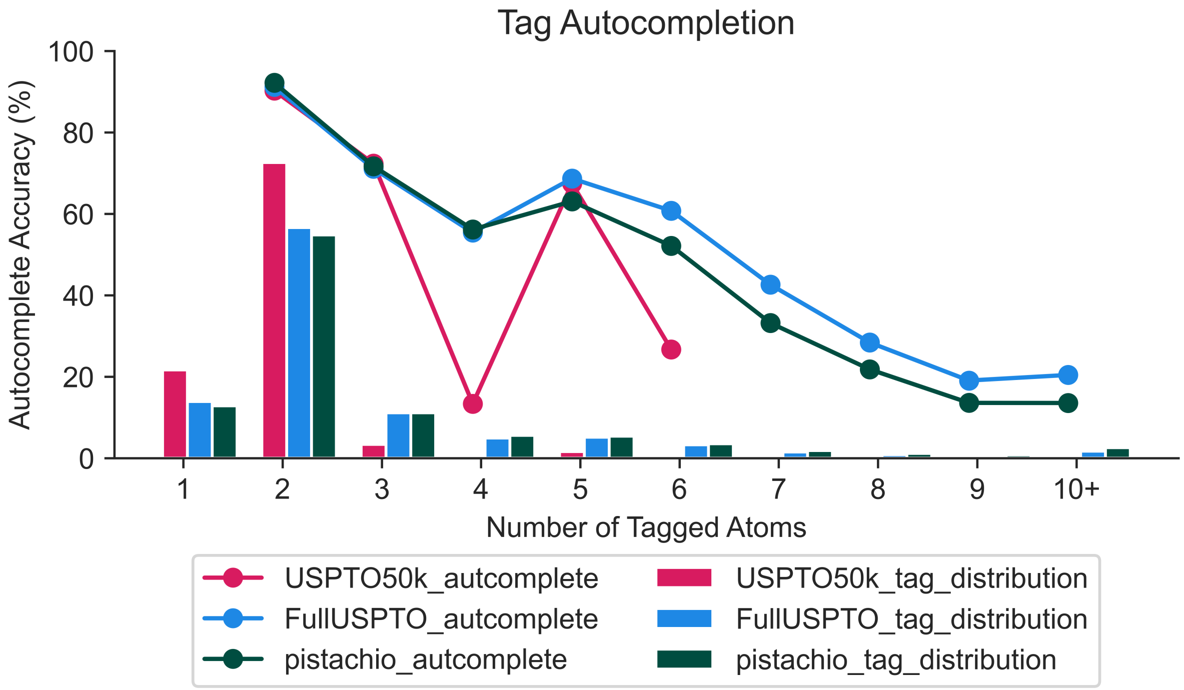

Figure S2: Performance of the tag autocompletion model with respect to the abilty to reconstruct the disconnection sites, shown for different sizes of the disconnection site as represented by the number of atom-tags. The ground-truth distribution of atom-tags for the respective datasets are shown. A correlation between the ability to reconstruct atom-tags and the training data is observed.

The *auto-complete tags* model achieved 77% accuracy on average across all datasets for tag reconstruction. Breaking it down by the number of tagged atoms and accuracy, the curve follows the same as for the tag distribution (Figure 2). We see the highest performance for number of tags equal to two, and a drop in performance for number of tags equals 4 as observed previously due to a lack of training data. Number of tags equal to one is omitted as it was not permuted, given that no permutations exist.

F. OpenNMT Model Training

The following command defines a transformer-based sequence-to-sequence model and trains it by optimizing the negative log-likelihood.

```
onmt_train -data $DATA -save_model $SAVE_MODEL -seed 42 -gpu_ranks 0 \
  -save_checkpoint_steps 5000 -keep_checkpoint 20 -train_steps 260000 \
  -param_init 0 -param_init_glorot -max_generator_batches 32 \
  -batch_size 6144 -batch_type tokens -normalization tokens \
  -max_grad_norm 0 -accum_count 4 -optim adam -adam_beta1 0.9 \
  -adam_beta2 0.998 -decay_method noam -warmup_steps 8000  \
  -learning_rate 2 -label_smoothing 0.0 -report_every 1000  \
  -valid_batch_size 8 -layers 4 -rnn_size 384 -word_vec_size 384 \
  -encoder_type transformer -decoder_type transformer -dropout 0.1 \
  -position_encoding -share_embeddings -global_attention general \
  -global_attention_function softmax -self_attn_type scaled-dot \
  -heads 8 -transformer_ff 2048
```

G. Trained Models, Metrics, and Experiments

Figure 3 has been reproduced from the manuscript to aid in interpretation of the results contained herein.

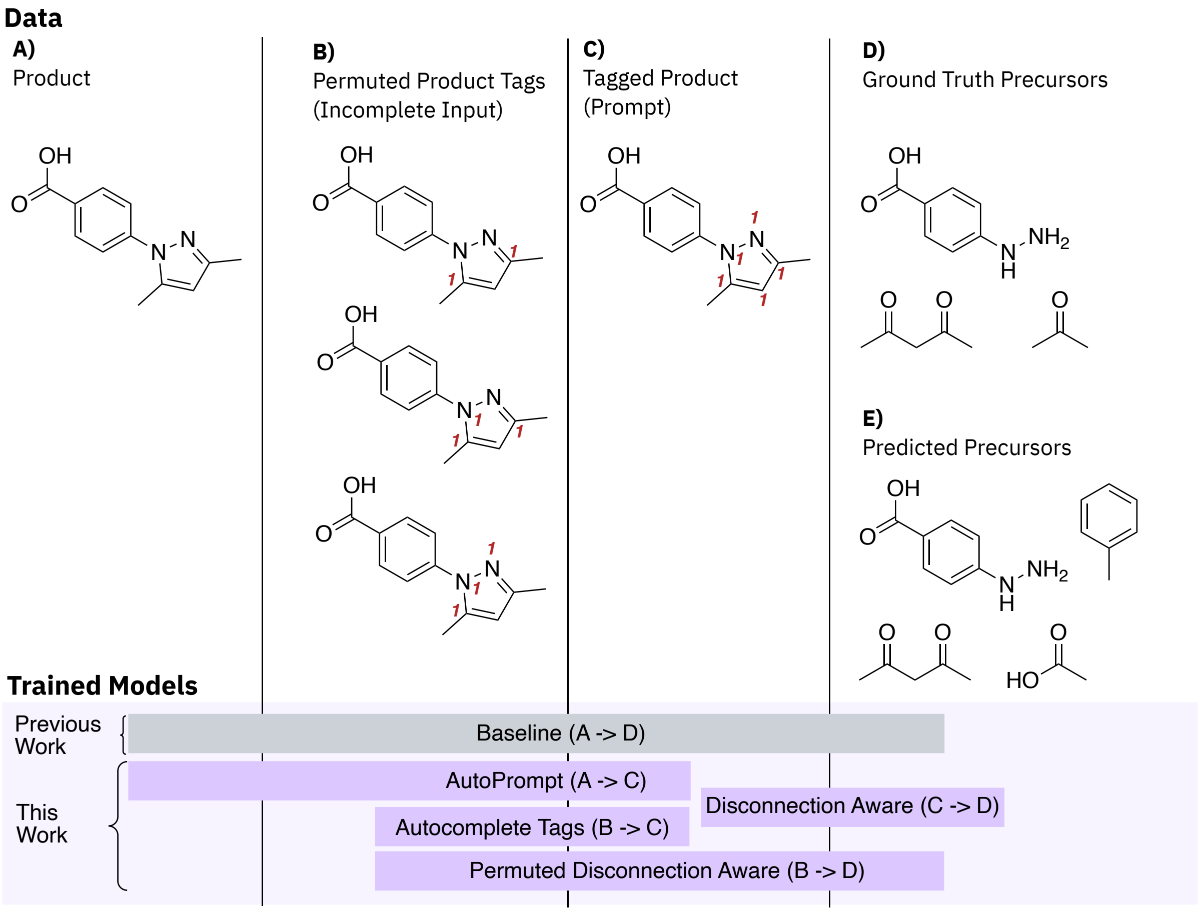

Figure S3: Overview of the experiments conducted and models trained illustrated with an example for a heterocycle formation.

G.1. Models

Table S3: Models and experiments ranked with respect to disconnection accuracy across the datasets examined. All values are quoted as an average across the number of atom tags. Alphabetical references to the training data are given as in 3.

Dataset	Model	In	Out	Invalid Precursors (%)	Matches Precursors (%)	Round Trip Acc (%)	Disconnection Acc (%)
ECReact (Enzyme)	Disconnection Aware	C	D	1.61	51.78	52.22	78.99
FullUSPTO	Disconnection Aware	C	D	1.06	14.15	49.92	72.59
Pistachio	Disconnection Aware	C	D	1.24	11.91	44.67	68.97
USPTO50k	Disconnection Aware	C	D	16.13	12.66	57.66	67.51
USPTO50k	Permuted Disconnection Aware	B	D	5.09	8.53	57.58	54.79
FullUSPTO	Permuted Disconnection Aware	B	D	0.87	11.47	61.16	50.12
Pistachio	Permuted Disconnection Aware	B	D	1.06	8.98	51.96	43.67
USPTO50k	Baseline	A	D	4.37	6.6	63.15	36.19
FullUSPTO	Baseline	A	D	0.66	9.35	80.02	33.86
Pistachio	Baseline	A	D	0.64	7.77	80.56	30.49

G.2. Exclusion of Reagents

Table S4: Experiments conducted where reagents where excluded from the training process show marginal differences as compared to the *disconnection aware* model including reagents.

Dataset	Invalid Precursors (%)	Matches Precursors (%)	Round Trip Acc (%)	Disconnection Acc (%)
FullUSPTO	1.27	40.54	43.88	73
USPTO50k	7.52	49.49	61.27	72.48
Pistachio	0.93	38.83	39.94	72.13

G.3. Incomplete Prompt Experiments

Table S5: Experiments conducted to determine the approach to take when presented with incomplete specification of the disconnection site, either generated by a human or automatic tagging model. The *disconnection aware* model is able to handle incomplete specification of the disconnection site albeit with slightly lower disconnection accuracy.

Dataset	Experiment	Invalid Precursors (%)	Matches Precursors (%)	Round Trip Acc (%)	Disconnection Acc (%)
FullUSPTO	Expt-1: Direct Inference	0.94	11.12	61.5	43.21
Pistachio	Expt-1: Direct Inference	1.14	8.97	54.16	40.45
USPTO50k	Expt-1: Direct Inference	25.61	1.73	26.3	12.35
USPTO50k	Expt-2: Autocompletion	18.81	9.39	48.38	49.85
FullUSPTO	Expt-2: Autocompletion	1.41	11.83	51.92	47.77
Pistachio	Expt-2: Autocompletion	1.35	9.24	48.88	43.89
USPTO50k	Expt-3: Model for Permutations	5.09	8.53	57.58	54.79
FullUSPTO	Expt-3: Model for Permutations	0.87	11.47	61.16	50.12
Pistachio	Expt-3: Model for Permutations	1.06	8.98	51.96	43.67

G.4. Check Tolerance of Tags in Baseline

Table S6: The *baseline* 'Molecular Transformer' was not able to handle atom-tags.

Dataset	Inference Model	Invalid Precursors (%)	Matches Precursors (%)	Round Trip Acc (%)	Disconnection Acc (%)
FullUSPTO	baseline	12.97	0.23	3.44	5.62
Pistachio	baseline	10.09	0.22	3.57	4.96
USPTO50k	baseline	27.47	0.33	7.84	3.12

H. Improved Class Diversity

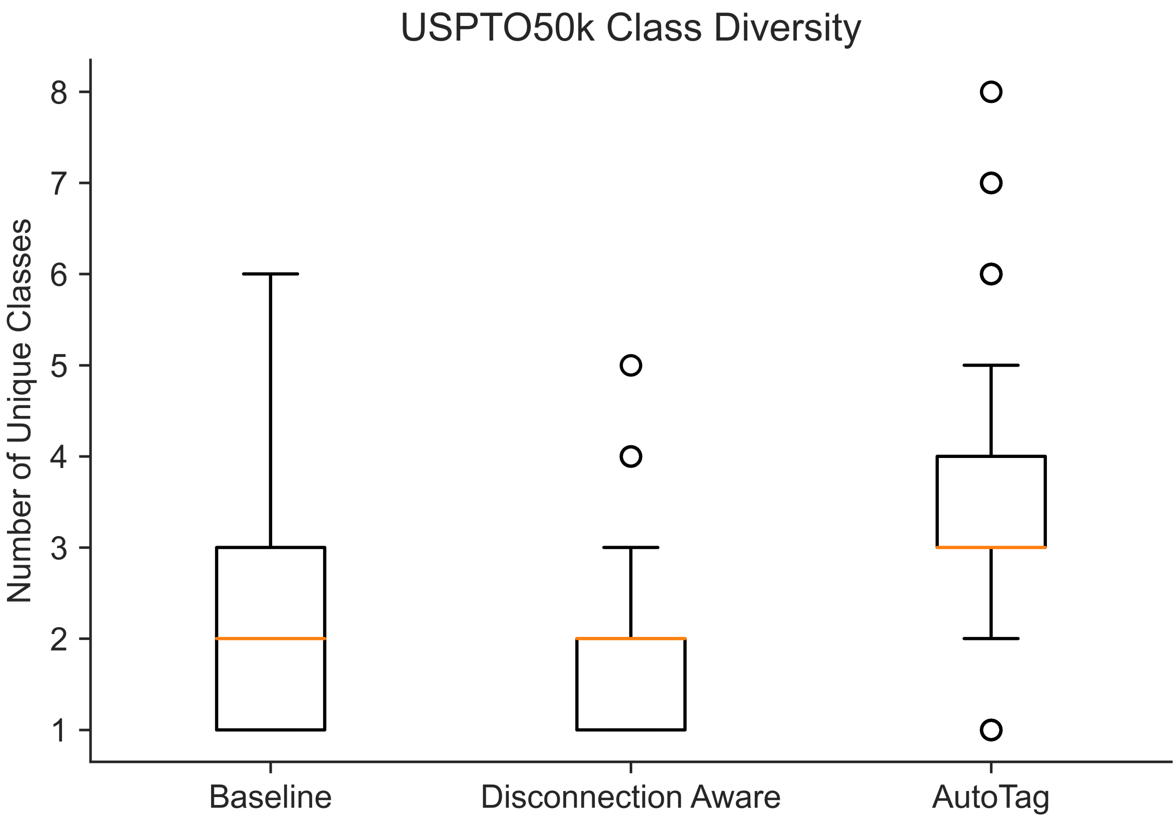

Figure S4: USPTO50k reaction class diversity

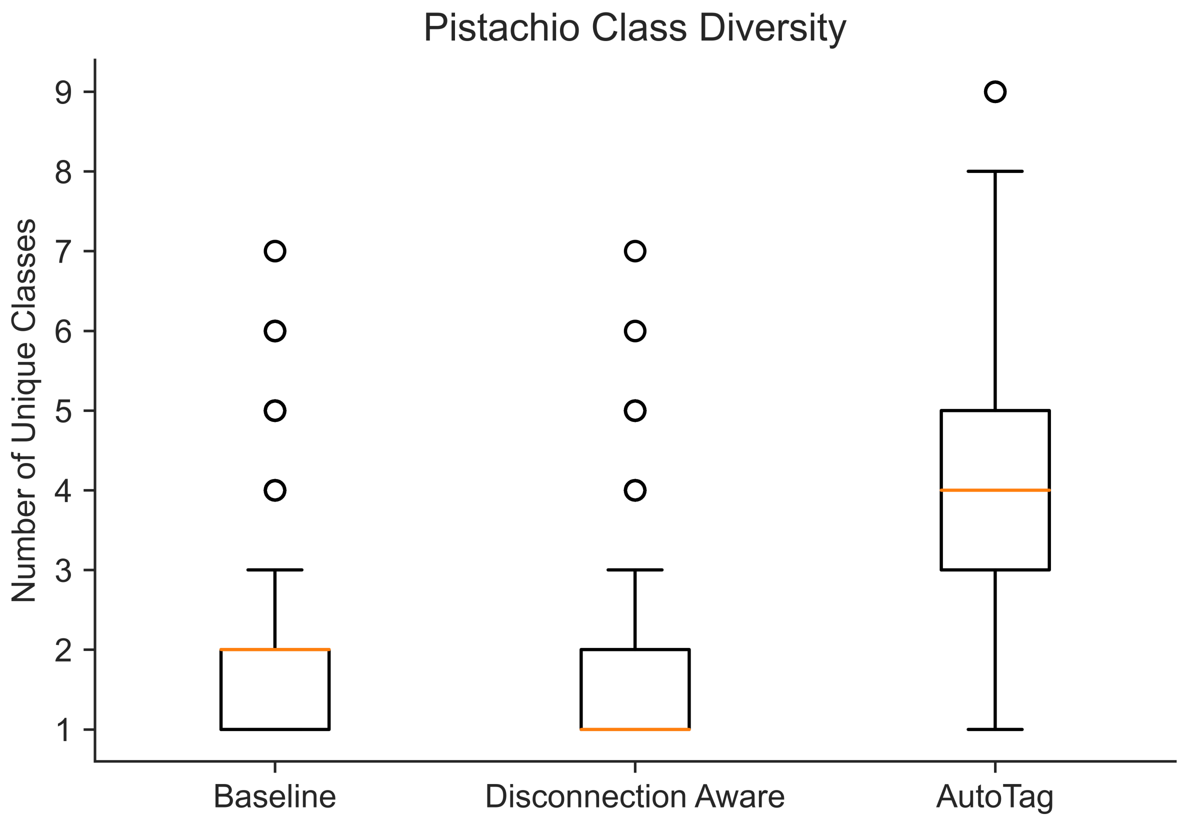

Figure S5: Pistachio reaction class diversity

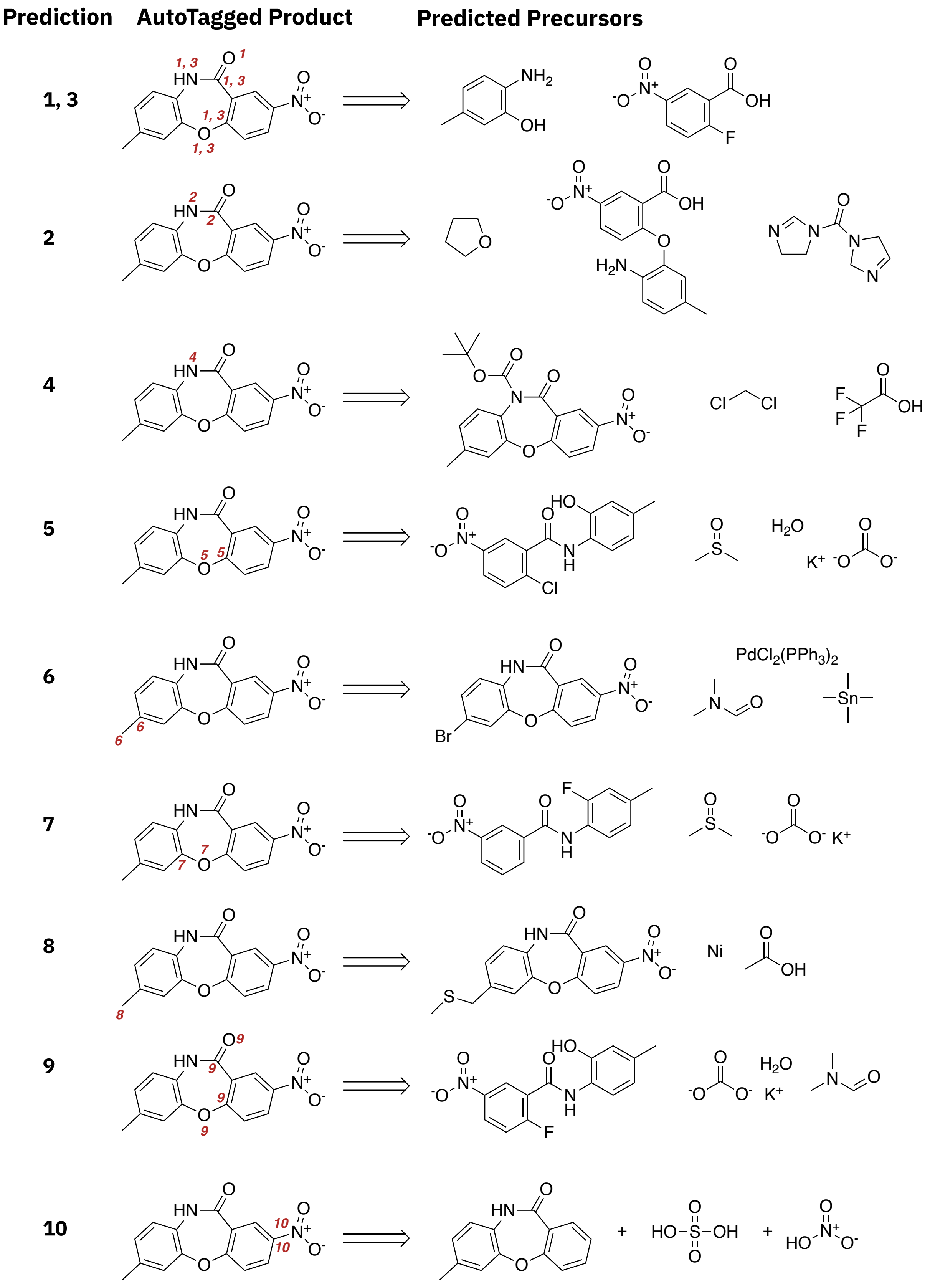

Figure S6: Complete set of predictions for the example given in the manuscript for the *AutoTag* model. The top10 predicted disconnection sites and associated precursors are shown.
